# A matched case-control study on the effectiveness of extracorporeal cytokine adsorption in critically ill patients

**DOI:** 10.1038/s41598-023-40719-z

**Published:** 2023-08-18

**Authors:** Alexander Jerman, Jakob Gubenšek, Jernej Berden, Vanja Peršič

**Affiliations:** 1 Department of Nephrology, University Medical Center Ljubljana, Ljubljana, Slovenia; 2 Department for Intensive Internal Medicine, University Medical Center Ljubljana, Ljubljana, Slovenia; 3 Faculty of Medicine, University of Ljubljana, Ljubljana, Slovenia

**Keywords:** Nephrology, Renal replacement therapy, Prognostic markers

## Abstract

Extracorporeal cytokine adsorption aims to reduce cytokine levels in critically ill patients. However, little convincing data exist to support its widespread use. This retrospective study compared interleukin-6 (IL-6) levels in patients treated with or without cytokine adsorber (CytoSorb®). Intensive care patients between Jan 2017 and Dec 2021 who had at least two IL-6 measurements were included. They were divided into an adsorber group and a standard of care group. We screened 3865 patients and included 52 patients in the adsorber group and 94 patients in the standard of care group. Matching was performed and the groups were compared regarding IL-6, lactate, CRP, procalcitonin, vasopressor requirement, and mortality rate. After matching, there were 21 patients in each group. Patients had similar age, ECMO and renal replacement therapy use, baseline noradrenaline requirement, serum lactate, pH, CRP, and IL-6 levels. There were no significant differences in the time course of IL-6, lactate, CRP, procalcitonin and noradrenaline requirement between groups. Two-day and ICU mortality and Kaplan-Meier estimated survival were also comparable. In this matched case-control study no difference in IL-6, inflammatory parameters, noradrenaline requirement or mortality was observed between patients treated with adsorber or standard of care.

## Introduction

Extracorporeal cytokine adsorption therapy remains controversial. The underlying premise for the use of extracorporeal cytokine adsorption is to reduce elevated levels of cytokines during a “cytokine storm” and the theoretical concepts of this therapy are very nicely laid out in Honore et al.^1^. Cytokine storm was historically poorly defined, with a recent update^2^. Despite the fact that high levels of cytokines are associated with mortality^3–5^, it is not clear whether or in which patients and/or in which clinical setting extracorporeal lowering of serum cytokine levels can improve survival^6^. Despite very promising conceptual idea, technologically impressive design (enormous adsorption area with strong, concentration-dependent adsorption and hemocompatibility^7^), apparent lack of serious adverse effects and relatively widespread use in some centers, the amount of data from controlled studies, showing efficiency of IL-6 removal and clinical improvement is very scarce. Nevertheless, a number of clinicians believe (based on personal observations and a number of published case-reports) that high cytokine levels (or even a single value of a single cytokine, e.g. interleukin-6 (IL-6)) are an indication for extracorporeal cytokine removal. A step forward was made recently with the proposal of a “dynamic scoring system (DSS)” to support the prescription of adsorption therapy^8^, not without a strong critique from peer-experts^9^. Altogether, the indications and/or patient selection are not yet clear.

In vitro data on cytokine reduction are excellent: the removal of cytokines is large, e.g. 99.6% for IL-6^10^. However, at baseline concentration of only 500–1000 pg/mL and without concomitant production, as is the case in vitro, it is difficult to extrapolate these results to the clinical setting. There are many case reports and uncontrolled case series, which cannot avoid bias, because the natural course of most cytokines is a rapid decrease in the absence of a trigger^11,12^. Furthermore, there is a well-known higher long-term mortality after severe sepsis, possibly due to immunosuppression^13^ and cytokine adsorption could additionally enhance these immunosuppressive effects. Research on this latter aspect is only in a development phase^14^. A number of randomized control trials^15–25^ and two recent meta analyses^26,27^ failed to establish a benefit for treated patients. There are few reports of potential adverse effects**.** Given the high number of procedures performed to date, it is unlikely that serious short-term adverse effects would be overlooked. However, the high variability of published data might easily hide more subtle problems. The removal of other non-toxic medium-to-high molecular weight molecules including antibiotics, non-antibiotic drugs and nutrients can be a problem but is rarely reported^28–31^.

In this study, we aimed to compare the time-course of IL-6 in patients treated with or without hemoadsorption and to compare clinical adsorber efficiency in terms of hemodynamic stability and mortality with standard treatment. We hypothesized that there is no significant difference in serum IL-6 levels and clinical course (lactate and noradrenaline requirement) between the groups.

## Methods

### Study design

This was a single-center, retrospective, matched, case-control study. Patients treated in the medical intensive care unit (ICU) of University Medical Center Ljubljana, Slovenia, were screened for inclusion. Effectiveness of cytokine adsorption therapy in terms of IL-6 removal and noradrenaline requirement was compared to standard of care (SOC). Baseline parameters that were different between preliminary groups were used as confounders for matching. Matching with genetic matching method was used to simulate randomization, and matching success was assessed in with the use of standardized mean differences (SMD). Patient data (demographics, clinical and laboratory parameters) were collected from hospital electronic health records. The laboratory results were determined using standard chemistry test and IL-6 measurements were made using the electrochemiluminescence assay (Cobas e 411, Roche Diagnostics GmbH, Mannheim, Germany). We calculated baseline SAPS II score, gathered data regarding renal replacement therapy (RRT), the use of extracorporeal membrane oxygenation (ECMO), mean arterial pressure (MAP) and norepinephrine/noradrenaline requirement at “time zero” (T0) and 4, 8, and 12 hours afterwards. When RRT was used at the same time as the adsorber, it was always connected serially, with CytoSorb cartridges placed in pre-filter position. CytoSorb was coupled with continuous veno-venous hemodialysis (CVVHD) with the ST100 filter in patients with severe hemodynamic instability or requirement for sustained fluid removal. CytoSorb was coupled with intermittent hemodialysis (IHD) with a synthetic high-flux dialyzer in patients with severe hyperkalemia, severe metabolic acidosis or hyperammonemia. Two-day and ICU mortality was recorded. The study complied with the Declaration of Helsinki (as revised in Tokyo 2013) and the National medical ethics committee approved the study (No. 0120-533/2019/5). Due to the retrospective nature of the study, the need for informed consent was waived.

### Study population

Patients treated between 01.01.2017 and 31.12.2021 in the medical ICU were included if they met the following criteria: (a) age > 18 years, (b) at least two IL-6 measurements, (c) if the patient was not treated with adsorption, there should be at least one IL-6 measurement after the maximum IL-6 value, (d) the patient was not treated with tocilizumab or an adsorber other than CytoSorb, (e) sufficient data for per-protocol analysis (laboratory data available 24 hours before or 48 hours after T0; for T0 definition see below). Patients, transferred out of the ICU were censored for survival. Patients were divided in two groups based on treatment received: the adsorber group (patients treated with adsorber (CytoSorb) for at least 3 hours) and the SOC group.

### Outcome measures

The primary outcome was the reduction in serum IL-6 levels and secondary outcomes were reduction of approximated values of serum IL-6, reduction of noradrenaline requirement, 2-day and ICU mortality. In the adsorber group, we defined “time zero” (T0) as the time of IL-6 measurement closest to the start of adsorption therapy (within – 10 to + 4 hours), while in the SOC group, T0 was defined as the peak IL-6 value. Other available IL-6 measurements were assigned to the nearest time point: 4, 8, 12, 16, and 20 hours after T0. Due to short IL-6 half-life, we chose a time interval of 4 hours to compare the elimination/reduction of laboratory values.

### Missing data extrapolation

IL-6 has a short half-life in the absence of persistent stimuli. However, frequent measurements are neither feasible nor economical in everyday clinical practice and, in addition, measurements rarely follow precise time intervals. Therefore, to increase the number of data points in an extended analysis, we extrapolated missing values of IL-6 in the following manner: we used a simplistic modeling of IL-6 elimination as first-order kinetics; case-reports^32,33^ generally support this simplification. We estimated missing data points using exponential curve extrapolation based on available IL-6 measurements. Each pair of available data points was used to calculate in-between missing data points. The first and last two points were also used to calculate marginal points.

### Statistical analysis

Baseline parameters were analyzed with Shapiro test for normality, and all were non-normally distributed. Between group differences were compared using Wilcoxon rank-sum test for continuous data and Chi-squared test for categoric data. Genetic matching with a 1:1 ratio without replacement was used to create two matched cohorts. Confounders for matching were selected according to baseline differences. The process was iteratively repeated until a reasonable number of matched pairs with acceptable residual standardized mean differences between groups was achieved. Because matching algorithm requires all data to be non-missing, missing values for selected confounders (8 for BMI, 6 for procalcitonin and 4 for CRP) were replaced with our population median value. The IL-6 levels and noradrenaline requirement were compared between groups using the Wilcoxon test for each selected time point. We considered and reported statistical significance at p < 0.05; we did not use multiple comparison correction. Mortality was assessed as a categorical parameter using Chi-squared test. Furthermore, Kaplan-Meier survival curves were compared with Log-rank test. Data were processed and analyzed using R and the following packages: tidyverse (processing), ggplot (plotting), MatchIt, matching, and cobalt (matching), survival, and survminer (survival analysis)^34^.

### Ethics approval and consent to participate

The study complied with the Declaration of Helsinki (as revised in Fortaleza in 2013). The Slovenian national ethics board (*Komisija Republike Slovenije za medicinsko etiko*, No. 0120-533/2019/5) approved the study and waived the need for the informed consent due to the retrospective nature of the study.

## Results

### Baseline and matched demographic data

**Figure 1:**
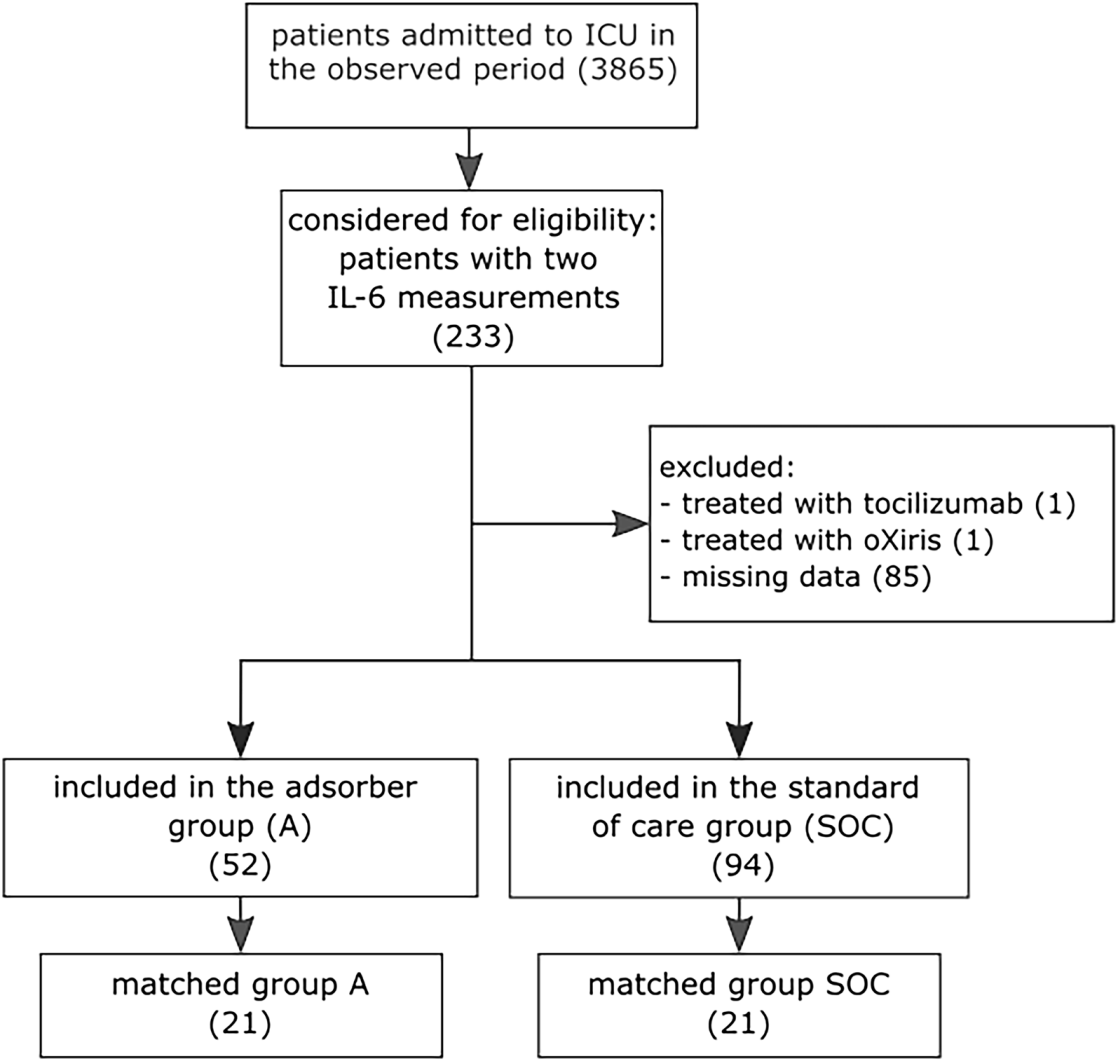
Patient selection and matching. Figure shows the working flow for patient selection and matching.

Patient selection is presented in Fig. 1. We screened 3865 patients treated in ICU during the observation period and included 52 patients in the adsorber group and 94 patients in the SOC group. Patients were excluded if they had missing electronic health records (1 patient), were treated with tocilizumab (1 patient) or oXiris® (1 patient), had inadequate laboratory data (17 patients), or had missing IL-6 data after T0 (1 patient in the adsorber group and 66 in the SOC group). After matching, each group included 21 patients.

**Figure 2:**
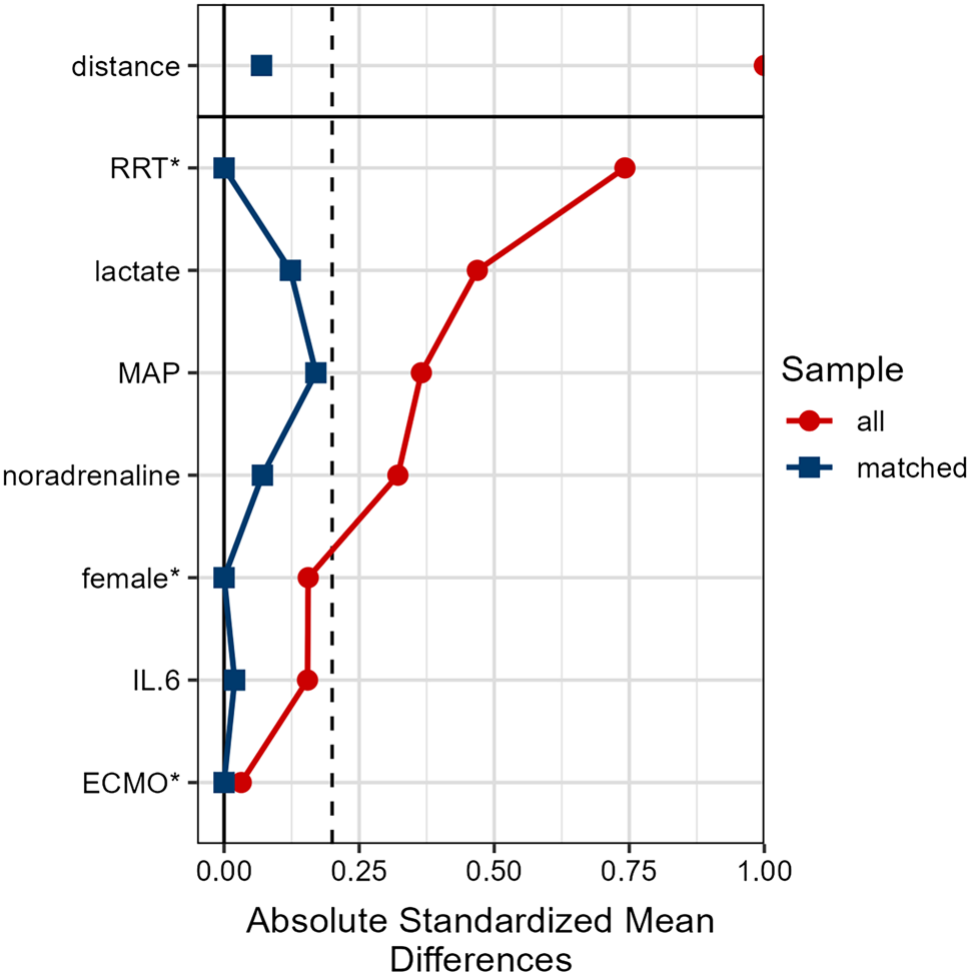
Covariate balance before and after matching. Figure shows which variables were selected for matching process, their absolute/raw standardized mean differences before and after the matching process. *Indicates variables for which the displayed value is the raw (unstandardized) difference in means.

Demographic data for unmatched groups are shown in Table 1 (left panel). The groups differed in sex distribution, requirement for RRT, IL-6, lactate and vasopressor dose. The underlying diseases were comparable, and the leading diagnosis was septic shock. Confounders selected for matching were based on differences between groups: sex, ECMO, RRT, MAP, noradrenaline requirement, serum lactate and IL-6 at T0. After matching, only lactate and MAP had a standardized mean difference (SMD) greater than 10% (12% and 17%, respectively). The mean for lactate was 7.9 vs. 7.3 mmol/L, and mean for MAP was 53.4 vs. 55.2 mmHg, for the adsorber and SOC groups, respectively (see Fig. 2). The matched patients’ characteristics (medians) were compared and are shown in Table 1 (right panel); there were no significant differences between groups.

**Table 1:** Baseline patient characteristics and mortality.

	Unmatched groups			Matched groups		
	Adsorber group, N = 52^1^	SOC group, N = 94^1^	p-value^2^	Adsorber group, N = 21^1^	SOC group, N = 21^1^	p-value^2^
Age (years)	57 (50, 68)	59 (44, 65)	0.7	53 (41, 68)	61 (56, 67)	0.2
Sex			**0.027**			>0.9
Female	7 (13%)	28 (30%)		5 (24%)	5 (24%)	
Male	45 (87%)	66 (70%)		16 (76%)	16 (76%)	
BMI (kg/m^2^)	27.8 (26.1, 31.3)	27.8 (24.3, 31.2)	0.3	29.4 (27.8, 32.7)	31.0 (27.0, 33.3)	0.9
Mortality						
2-day	18 (35%)	24 (26%)	0.2	7 (33%)	8 (38%)	0.7
ICU	28 (54%)	40 (43%)	0.2	12 (57%)	13 (62%)	0.8
Diagnosis			0.074			>0.9
IHCA	1 (1.9%)	8 (8.6%)		0 (0%)	1 (4.8%)	
Intoxication	4 (7.7%)	1 (1.1%)		0 (0%)	0 (0%)	
OHCA	10 (19%)	11 (12%)		3 (14%)	2 (9.5%)	
Other	2 (3.8%)	10 (11%)		1 (4.8%)	2 (9.5%)	
Shock - other	13 (25%)	19 (20%)		7 (33%)	6 (29%)	
Shock - sepsis	22 (42%)	44 (47%)		10 (48%)	10 (48%)	
ECMO	14 (27%)	28 (30%)	0.7	5 (24%)	5 (24%)	>0.9
RRT	52 (100%)	24 (26%)	**<0.001**	21 (100%)	21 (100%)	
MAP (mmHg)	55 (48, 63)	58 (50, 65)	0.10	49 (48, 63)	55 (50, 59)	0.4
SAPS II	82 (69, 96)	79 (63, 88)	0.12	83 (71, 90)	85 (76, 92)	0.5
Noradrenaline (mg/kg/h)	0.036 (0.025, 0.051)	0.028 (0.010, 0.046)	**0.038**	0.04 (0.01, 0.05)	0.03 (0.01, 0.04)	0.8
Vasopressin	12 (23%)	13 (14%)	0.2	5 (24%)	5 (24%)	>0.9
Adrenaline	2 (3.8%)	3 (3.2%)	>0.9	1 (4.8%)	0 (0%)	>0.9
Dobutamine	17 (33%)	28 (30%)	0.7	6 (29%)	7 (33%)	0.7
Any additional vasopressor	24 (46%)	36 (39%)	0.4	9 (43%)	9 (43%)	>0.9
Lactate (mmol/L)	6.8 (3.4, 10.9)	3.6 (1.5, 7.3)	**<0.001**	6.8 (4.5, 10.9)	5.4 (2.4, 10.6)	0.5
IL-6 (ng/L)	2,174 (880, 14,502)	915 (300, 3,870)	**0.003**	2,441 (1,169, 27,968)	2,552 (262, 18,680)	0.6
Procalcitonin (µg/L)	10 (2, 38)	7 (1, 24)	0.094	20 (6, 59)	11 (3, 41)	0.5
Creatinine (µmol/L)	197 (145, 318)	174 (103, 276)	0.11	255 (192, 340)	269 (215, 386)	0.7
eGF^3^ (ml/min/1.73 m^2^)	31 (19, 49)	39 (20, 65)	0.2	24 (15, 32)	18 (14, 30)	0.5
CRP (mg/L)	163 (42, 253)	163 (47, 256)	>0.9	182 (110, 247)	167 (62, 254)	0.7
pH	7.30 (7.23, 7.36)	7.30 (7.21, 7.41)	0.4	7.27 (7.25, 7.36)	7.21 (7.19, 7.31)	0.2

Data presented as median (IQR) or number (percent).

*ECMO* extracorporeal membrane oxygenation, *RRT* renal replacement therapy (in 12h), *MAP* mean arterial pressure, *SAPS* II simplified acute physiology score 2.

^1^Median (IQR); n (%).

^2^Wilcoxon rank sum test; Chi-squared test.

^3^eGF – estimated glomerular filtration, CKD-EPI Creatinine 2021.

Significant values are in bold.

### Changes in IL-6, lactate, procalcitonin and CRP in matched groups

**Figure 3:**
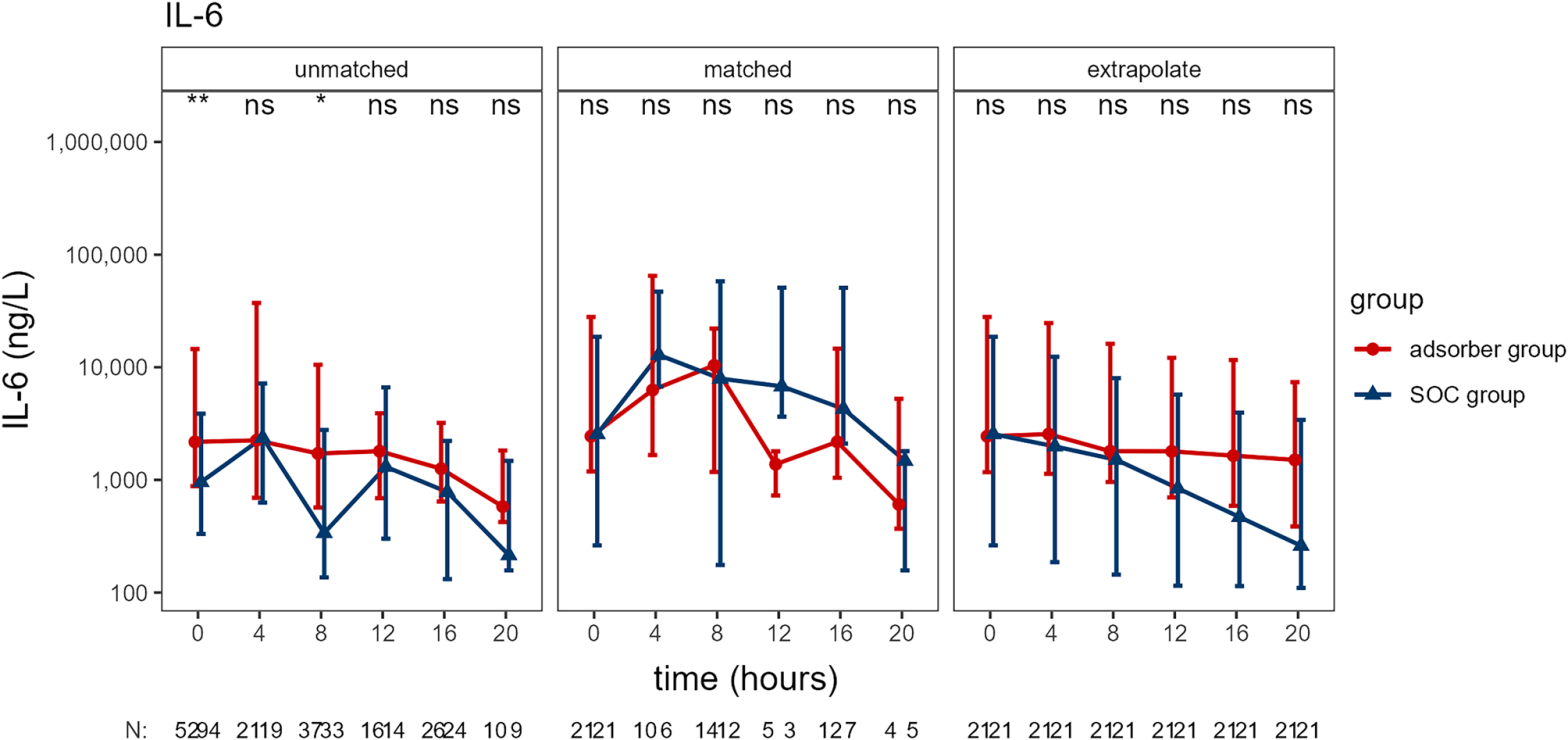
Interleukin-6 time course in unmatched and matched groups, and extrapolated data for matched group. The time course of serum IL-6 is shown. “N” indicates number of available data entries at each time point, this is only complete (N = 21) for all cases in “extrapolate” group, see “Methods” for the calculation. Data presented as median and IQR. *p ≤ 0.05, **p ≤ 0.01.

**Figure 4:**
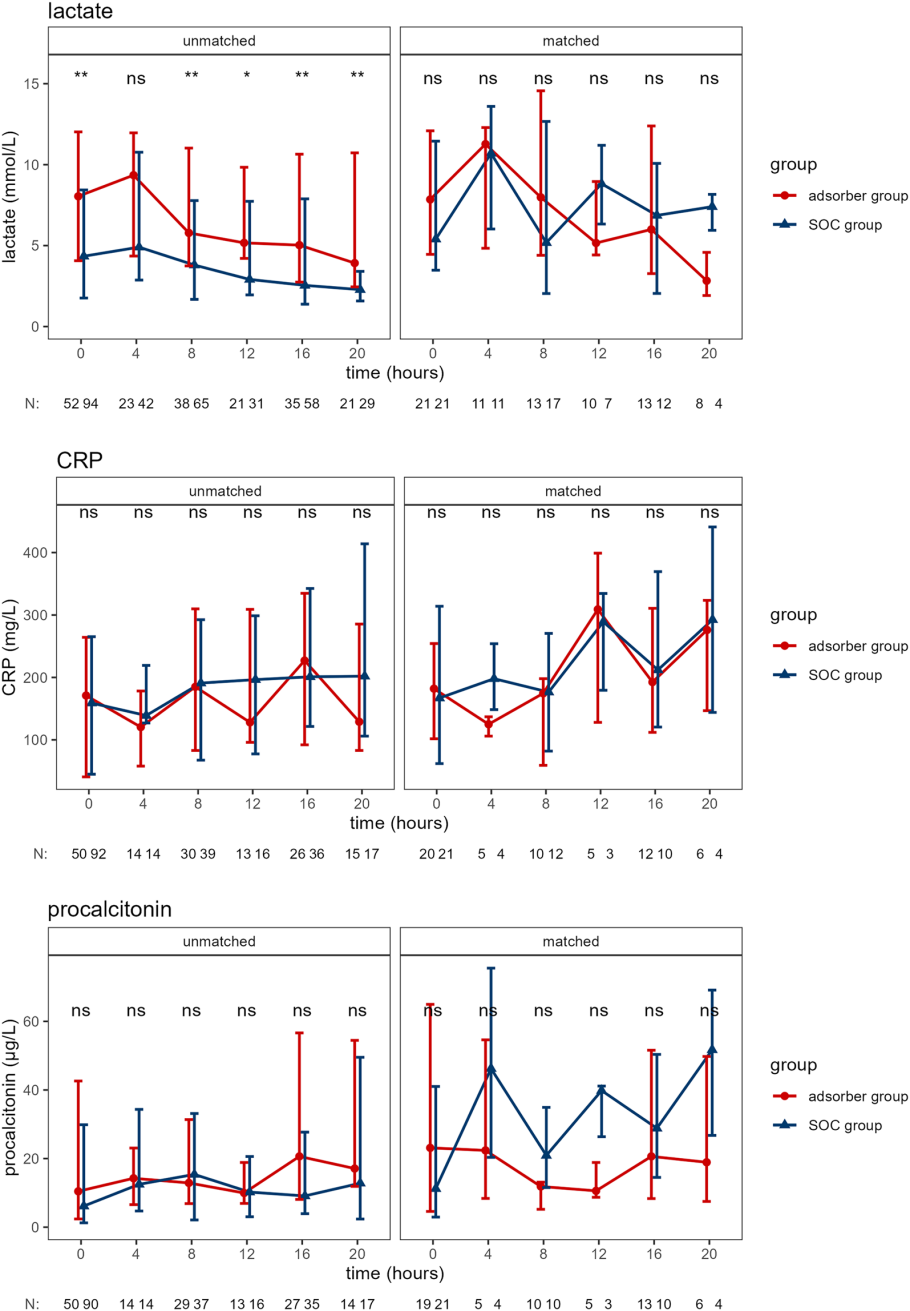
Serum lactate, CRP, and procalcitonin in unmatched and matched groups. The time course of serum lactate, CRP, and procalcitonin is shown. “N” indicates number of available data entries at each time point. Data presented as median and IQR. *p ≤ 0.05, **p ≤ 0.01.

Time course of IL-6 differed only slightly in unmatched groups. However, after matching the differences were no longer significant. Furthermore, after performing extrapolation for missing time points in the matched groups, the time course was even more similar (see Fig. 3). Serum lactate as a marker of shock severity was different between the groups: patients treated with adsorber had higher levels. After matching reasonable similarity was achieved at baseline and the time course showed a similar pattern, without significant differences at any time point. Furthermore, CRP and procalcitonin time course did not differ significantly between the groups (see Fig. 4).

### Vasopressor requirement in matched groups

**Figure 5:**
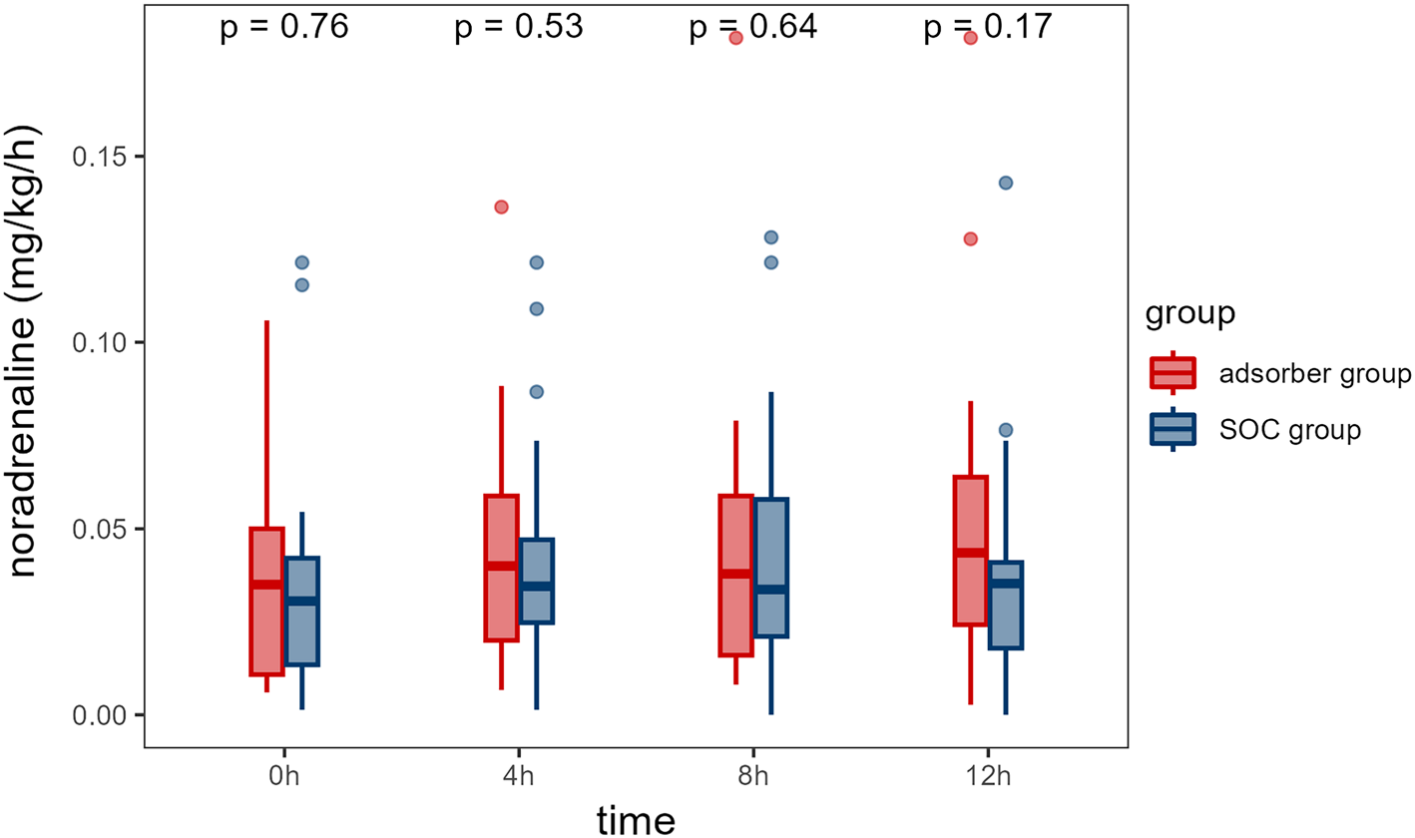
Noradrenaline requirement in matched pairs. Noradrenaline requirement (mg/kg/h) for matched pairs at different time points. Differences between the groups are calculated with Wilcoxon test for each selected time point, p value is shown.

Noradrenaline requirement was comparable in matched groups at baseline (by design) and absolute doses of noradrenalin were low. In the following 12 hours, there were no significant differences within groups at predefined time points (Table 2, Fig. 5) and also compared with baseline (Table S2). There was a slight, nonsignificant increase in noradrenaline requirement after 4 hours in the adsorber group, which seemed to persist.

**Table 2:** Noradrenaline requirement (in mg/kg/h) in matched groups.

Time (h)	Adsorber group, N = 21^1^	SOC group, N = 21^1^	p-value^2^
0	0.035 (0.011, 0.050)	0.031 (0.013, 0.042)	0.763
4	0.040 (0.020, 0.059)	0.035 (0.025, 0.047)	0.529
8	0.038 (0.016, 0.059)	0.034 (0.021, 0.058)	0.642
12	0.044 (0.024, 0.064)	0.035 (0.018, 0.041)	0.173

Data presented as median (IQR) per period.

^1^Median (IQR).

^2^Wilcoxon rank sum test.

### Mortality

**Figure 6:**
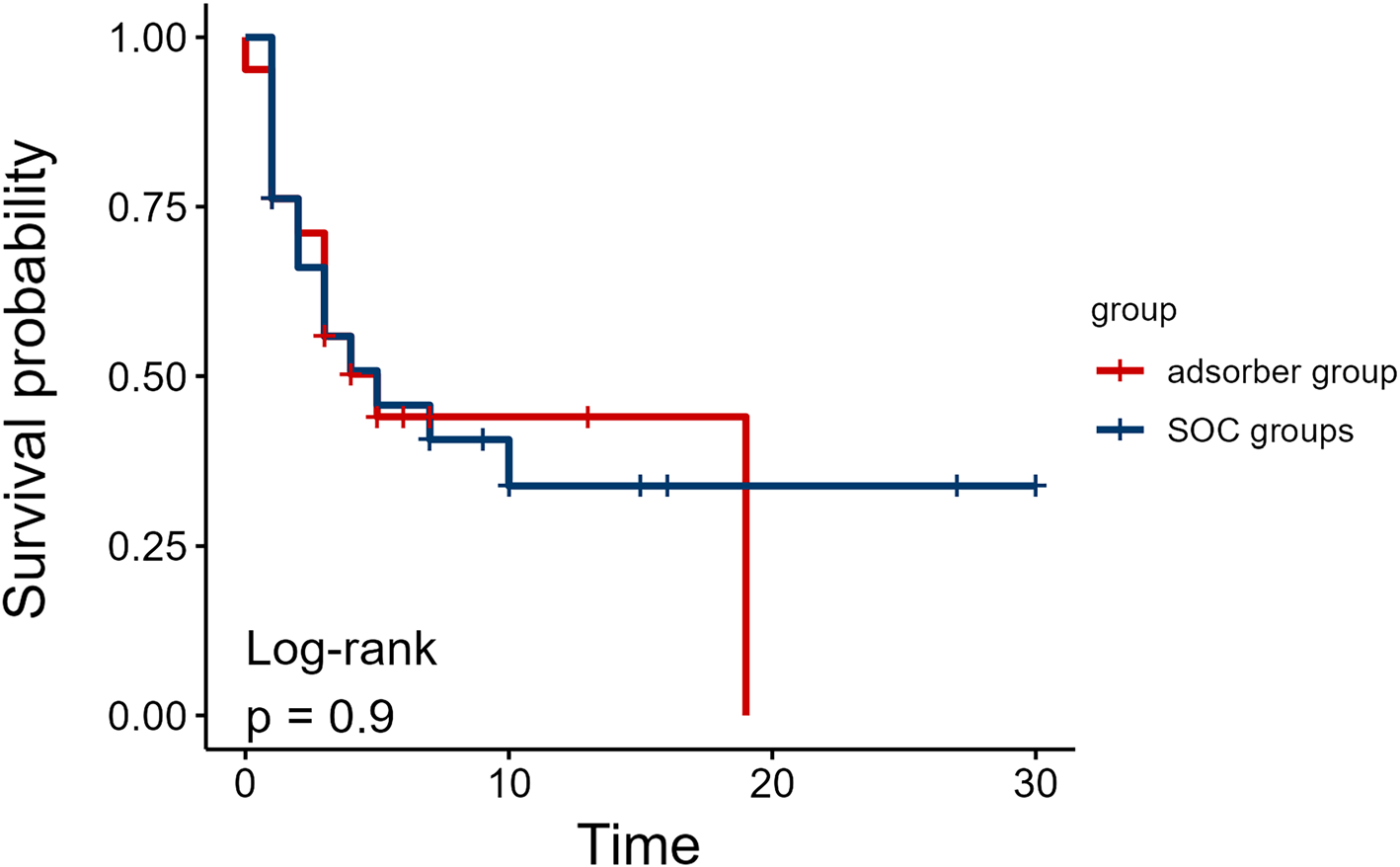
Kaplan-Meier estimated survival probability in matched groups. Survival probability between matched groups was estimated with Kaplan-Meier survival curves and compared with the Log-rank test, p value is shown.

Two-day and ICU mortality did not differ between groups (Table 1). Kaplan-Meier estimated survival in the first 30 days was also comparable between groups (Log-rank test p = 0.9, Fig. 6). Stratification of matched patients by mortality revealed higher baseline IL-6, higher lactate and higher IL-6 at 12 hours, but lower CRP at 12 hours in the nonsurvivor group (Table S1).

## Discussion

In this study, we retrospectively analyzed the time course of IL-6 in patients treated with CytoSorb adsorber or SOC and found that in the matched groups the time course was comparable. There were also no differences in lactate, noradrenaline requirement, inflammatory markers or mortality.

Elevated levels of IL-6 in conjunction with appropriate clinical settings are usually interpreted as a ‘cytokine storm’. However, it is unclear which cytokines are the best markers of sepsis associated cytokine storm^2^. We chose IL-6, which is the most commonly used and has also been implemented into our local clinical practice. Measurement of other cytokines, such as TNF-alpha or IL-1, could be more suitable for sepsis-associated hyperinflammation. There is an association between high IL-6 levels and mortality^3,4^ as well as the need for organ-supportive therapies^5^. We also found that IL-6 and lactate were significantly lower in survivors at 12 hours (Table S1), while Weidhase^35^ also observed lower CRP and procalcitonin. In line with these findings, removal of IL-6 by extracorporeal adsorption techniques seems to be a reasonable therapeutic option. A recent consensus report on acute kidney injury associated with sepsis did not grade the use of adsorption therapies but suggested that such approaches could be considered in patients meeting clinical and biological criteria^36^. It is well known that in the absence of a triggering signal (DAMP/PAMP), cytokines levels spontaneously decrease very rapidly^32,37^ and that lower serum IL-6 levels at 48-72 hours are associated with successful antibiotic therapy^35^. Therefore, in the absence of positive randomized studies there is no definite answer as to whether extracorporeal elimination of cytokines truly contributes to improved outcome of patients with cytokine storm^6^.

Our study was conducted as a replication of the study by Scharf et al.^38^. However, due to slightly different patient selection, the results are not directly comparable. Our matched cohorts had similar age, sex, BMI, 2-day mortality, SAPS II score, CRP and proportion of ECMO patients; additionally, patients in both cohorts were treated with RRT. The fact that all patients in matched cohorts were treated with RRT removes any potential bias due to additional treatment effect of RRT itself. A considerable number of patients were treated with ECMO, similar to Scharf’s study^38^. In their study, patients were also more ill at baseline: noradrenaline requirement was higher (median 3.8 and 3.6 vs 3.2 and 2.8 mg/h, serum lactate was also higher (median 7.8 and 8.2 vs 6.8 and 5.4 mmol/L), and IL-6 was much higher in Scharf's group (median 58,385 and 59,812 vs. 2,441 and 2,552 ng/L)^38^. Although comparable between groups, the noradrenaline requirement and serum lactate levels indicate that our patients (in general) were not at the high-end of the spectrum of hemodynamic instability. As reported previously, we tend to use Cytosorb relatively early, but only after a stabilization period, allowing for some patients to improve with SOC only^39^. It is noteworthy that a considerable proportion of our patients had an additional vasopressor, which is often used in ICU as a norepinephrine-sparing strategy. Taken altogether, although patients in Scharf’s group had more severe disease, the conclusions are similar. There was no survival advantage, no difference in the reduction of IL-6 and lactate and no difference in hemodynamic stabilization also in our cohort of less severely ill patients treated with the Cytosorb adsorber. Therefore, we cannot confirm that selecting patients according to severity of illness would be appropriate for the use of CytoSorb.

Because cytokine levels decrease spontaneously very rapidly in the absence of a triggering signal (DAMP/PAMP)^32,37^ and the reduction of IL-6 in our study was observed in both our groups (adsorber and SOC), the question arises as to the appropriateness of measuring serum IL-6 as a marker of clinically relevant improvement in patients with septic shock treated with hemoadsorption. To address this question, we additionally evaluated clinical efficacy of hemoadsorption in terms of hemodynamic stability, inflammatory markers and mortality. Early reports suggested lower vasopressor requirement^11,39,40^ with the use of CytoSorb. A randomized study reported vasopressor requirement reduction in patients treated with adsorber for 24 hours, but not in the control group^17^. A matched-controls study by Rugg et al.^41^ showed similar results with lower vasopressor requirements, but the groups differed significantly (p = 0.014) in terms of starting noradrenaline dose despite the matching process. In contrast, our study showed no benefit in terms of vasopressor reduction, as was also observed by other authors^42,43^. In addition, serum lactate and inflammatory markers (CRP and procalcitonin) also did not differ substantially.

Finally, mortality was also not different between our unmatched and matched groups. This is in contrast to our retrospective study, where lower observed versus predicted mortality in patients treated with CytoSorb was reported^39^ and in contrast to the propensity-score-weighted retrospective study, in which CytoSorb was associated with a decreased observed versus expected 28-day all-cause mortality compared with continuous renal replacement therapy alone^44^. On the other hand, our results are analogous to Scharf et al.^38^, where matched groups had similar 2-day and in-hospital mortality.

In our two matched groups, we found that CytoSorb hemoadsorption did not seem to provide a survival benefit compared with SOC. However, our study has several limitations. First, we did not report the time interval between ICU admission and the start of adsorber and SOC treatment. Some clinical reports describe rapid hemodynamic stabilization and better survival with early use of CytoSorb as an adjunctive therapy to SOC^41^, compared with SOC alone^8^. A recent report of successful treatment of septic shock patients with adjuvant therapeutic plasma exchange (TPE)^45^ also supports the need to »act fast«: in a small bicenter randomized trial, the use of TPE in the first 24 hours led to significant (p < 0.0001) noradrenaline reduction 6 hours after randomization. Additional studies should be performed to determine the characteristics of patients who might benefit from CytoSorb therapy and when is the optimal time to start adsorption therapy. Secondly, because of the lack of stringent criteria for adsorbent use, selection bias is always introduced in non-randomized studies. The selection of data introduced bias because only patients with a documented reduction in IL-6 levels were included in the control group. Furthermore, retrospective data should always be analyzed and interpreted with the greatest caution, but matching is one of the best methods for reducing group imbalances and provides a way to extract a hidden ‘quasi-randomized’ subpopulation from a larger observed population. Several matching methods were used in a comparable way to asses adsorber effectiveness (propensity-score matching, genetic matching, inverse propensity treatment weights)^38,41,44^. We selected genetic matching, which is a computational method combining benefits of Mahalanobis matching and propensity-score matching. Since the data were collected retrospectively our analysis suffered from a limited number of IL-6 measurements. As to strengthen our results, we used a model for IL-6 kinetics approximation.

## Conclusions

In conclusion, time course analysis of IL-6 in patients treated with CytoSorb or SOC was comparable in the matched groups and no differences in lactate, noradrenaline requirement, inflammatory markers or mortality between matched groups were confirmed. Additional prospective studies should therefore be designed to elucidate the role, timing and duration of adsorbent treatment in septic patients.

### Supplementary Information

Supplementary Tables.

## Data Availability

The datasets used and/or analyzed during the current study are available from the corresponding author on reasonable request.

## References

[1] Honore PM (2019). Cytokine removal in human septic shock: Where are we and where are we going?. Ann. Intensive Care.

[2] Fajgenbaum DC, June CH (2020). Cytokine storm. N. Engl. J. Med..

[3] Pinsky MR (1993). Serum cytokine levels in human septic shock. Relation to multiple-system organ failure and mortality. Chest.

[4] Patel RT, Deen KI, Youngs D, Warwick J, Keighley MR (1994). Interleukin 6 is a prognostic indicator of outcome in severe intra-abdominal sepsis. Br. J. Surg..

[5] Picod A (2022). Systemic inflammation evaluated by interleukin-6 or C-reactive protein in critically ill patients: Results from the FROG-ICU study. Front. Immunol..

[6] Mehta Y (2023). Extracorporeal blood purification strategies in sepsis and septic shock: An insight into recent advancements. World J. Crit. Care Med..

[7] Song M (2004). Cytokine removal with a novel adsorbent polymer. Blood Purif..

[8] Kogelmann K (2021). First evaluation of a new dynamic scoring system intended to support prescription of adjuvant CytoSorb hemoadsorption therapy in patients with septic shock. J. Clin. Med..

[9] Supady, A., Lepper, P. M., Duerschmied, D. & Wengenmayer, T. On the use of hemadsorption with CytoSorb in patients with septic shock. Comment on Kogelmann et al. First evaluation of a new dynamic scoring system intended to support prescription of adjuvant CytoSorb hemoadsorption therapy in patients with septic shock. J. Clin. Med., 10, 2939 (2021). *J. Clin. Med.*, **11**, 334 (2022). 10.3390/jcm11020334 PMC8781718 35054028

[10] Malard B, Lambert C, Kellum JA (2018). In vitro comparison of the adsorption of inflammatory mediators by blood purification devices. Intensive Care Med. Exp..

[11] Calabrò MG (2019). Blood purification with CytoSorb in critically ill patients: Single-center preliminary experience. Artif. Organs.

[12] Houschyar KS, Nietzschmann I, Siemers F (2017). The use of a cytokine adsorber (CytoSorb) in a patient with septic shock and multi-organ dysfunction (MODS) after a severe burn injury. Handchir. Mikrochir. Plast. Chir. Organ Deutschsprachigen Arbeitsgemeinschaft Handchir. Organ Deutschsprachigen Arbeitsgemeinschaft Mikrochir. Peripher. Nerven Gefasse Organ V.

[13] Quartin AA, Schein RM, Kett DH, Peduzzi PN (1997). Magnitude and duration of the effect of sepsis on survival. Department of Veterans Affairs Systemic Sepsis Cooperative Studies Group. JAMA.

[14] Bruse N, Leijte GP, Pickkers P, Kox M (2019). New frontiers in precision medicine for sepsis-induced immunoparalysis. Expert Rev. Clin. Immunol..

[15] Bernardi MH (2016). Effect of hemoadsorption during cardiopulmonary bypass surgery—A blinded, randomized, controlled pilot study using a novel adsorbent. Crit. Care Lond. Engl..

[16] Schädler D (2017). The effect of a novel extracorporeal cytokine hemoadsorption device on IL-6 elimination in septic patients: A randomized controlled trial. PLoS One.

[17] Hawchar F (2019). Extracorporeal cytokine adsorption in septic shock: A proof of concept randomized, controlled pilot study. J. Crit. Care.

[18] Poli EC (2019). Cytokine clearance with CytoSorb® during cardiac surgery: A pilot randomized controlled trial. Crit. Care.

[19] Wagner R (2019). Plasma levels of myocardial MicroRNA-133a increase by intraoperative cytokine hemoadsorption in the complex cardiovascular operation. J. Clin. Med. Res..

[20] Taleska Stupica G (2020). Extracorporeal hemadsorption versus glucocorticoids during cardiopulmonary bypass: A prospective, randomized, controlled trial. Cardiovasc. Ther..

[21] Asch S (2021). The effect of perioperative hemadsorption in patients operated for acute infective endocarditis—A randomized controlled study. Artif. Organs.

[22] Diab M (2022). Cytokine hemoadsorption during cardiac surgery versus standard surgical care for infective endocarditis (REMOVE): Results from a multicenter randomized controlled trial. Circulation.

[23] Supady A (2021). Cytokine adsorption in patients with severe COVID-19 pneumonia requiring extracorporeal membrane oxygenation (CYCOV): A single centre, open-label, randomised, controlled trial. Lancet Respir. Med..

[24] Stockmann H (2022). CytoSorb rescue for COVID-19 Patients with vasoplegic shock and multiple organ failure: A prospective, open-label, randomized controlled pilot study*. Crit. Care Med..

[25] Supady A (2022). Cytokine adsorption in patients with post-cardiac arrest syndrome after extracorporeal cardiopulmonary resuscitation (CYTER)—A single-centre, open-label, randomised, controlled trial. Resuscitation.

[26] Heymann M, Schorer R, Putzu A (2022). Mortality and adverse events of hemoadsorption with CytoSorb® in critically ill patients: A systematic review and meta-analysis of randomized controlled trials. Acta Anaesthesiol. Scand..

[27] Becker S (2023). Efficacy of CytoSorb®: A systematic review and meta-analysis. Crit. Care.

[28] Scharf C (2022). Does the cytokine adsorber CytoSorb® reduce vancomycin exposure in critically ill patients with sepsis or septic shock? A prospective observational study. Ann. Intensive Care.

[29] Schneider AG (2021). Pharmacokinetics of anti-infective agents during CytoSorb hemoadsorption. Sci. Rep..

[30] Dimski T, Brandenburger T, MacKenzie C, Kindgen-Milles D (2020). Elimination of glycopeptide antibiotics by cytokine hemoadsorption in patients with septic shock: A study of three cases. Int. J. Artif. Organs.

[31] Scheier J (2022). Mechanistic considerations and pharmacokinetic implications on concomitant drug administration during CytoSorb therapy. Crit. Care Explor..

[32] da Silva AMT (1993). Shock and multiple-organ dysfunction after self-administration of salmonella endotoxin. N. Engl. J. Med..

[33] Kuribayashi T (2018). Elimination half-lives of interleukin-6 and cytokine-induced neutrophil chemoattractant-1 synthesized in response to inflammatory stimulation in rats. Lab. Anim. Res..

[34] R: A language and environment for statistical computing. R Foundation for Statistical Computing (2022).

[35] Weidhase L (2019). Is interleukin-6 a better predictor of successful antibiotic therapy than procalcitonin and C-reactive protein? A single center study in critically ill adults. BMC Infect. Dis..

[36] Zarbock A (2023). Sepsis-associated acute kidney injury: Consensus report of the 28th Acute Disease Quality Initiative workgroup. Nat. Rev. Nephrol..

[37] Chousterman BG, Swirski FK, Weber GF (2017). Cytokine storm and sepsis disease pathogenesis. Semin. Immunopathol..

[38] Scharf C (2021). Can the cytokine adsorber CytoSorb® help to mitigate cytokine storm and reduce mortality in critically ill patients? A propensity score matching analysis. Ann. Intensive Care.

[39] Persic V (2022). Effect of CytoSorb coupled with hemodialysis on interleukin-6 and hemodynamic parameters in patients with systemic inflammatory response syndrome: A retrospective cohort study. J. Clin. Med..

[40] Kogelmann K, Jarczak D, Scheller M, Drüner M (2017). Hemoadsorption by CytoSorb in septic patients: A case series. Crit. Care Lond. Engl..

[41] Rugg C (2020). Hemoadsorption with CytoSorb in septic shock reduces catecholamine requirements and in-hospital mortality: A single-center retrospective ‘genetic’ matched analysis. Biomedicines.

[42] Mehta Y (2020). Experience with hemoadsorption (CytoSorb®) in the management of septic shock patients. World J. Crit. Care Med..

[43] Zuccari S (2020). Changes in cytokines, haemodynamics and microcirculation in patients with sepsis/septic shock undergoing continuous renal replacement therapy and blood purification with CytoSorb. Blood Purif..

[44] Brouwer WP, Duran S, Kuijper M, Ince C (2019). Hemoadsorption with CytoSorb shows a decreased observed versus expected 28-day all-cause mortality in ICU patients with septic shock: A propensity-score-weighted retrospective study. Crit. Care.

[45] David S (2021). Adjuvant therapeutic plasma exchange in septic shock. Intensive Care Med..

