# Supplementary material for: A matched case-control study on the effectiveness of extracorporeal cytokine adsorption in critically ill patients
Source: Sci Rep. 2023 Aug 18;13:13464. doi: 10.1038/s41598-023-40719-z (PMC10439174; doi:10.1038/s41598-023-40719-z)
Supplement: Supplementary file 1 — Supplementary Tables. [file 41598_2023_40719_MOESM1_ESM.docx]

# A matched case-control study on the effectiveness of extracorporeal cytokine adsorption in critically ill patients

Alexander Jerman, Jakob Gubenšek, Jernej Berden, Vanja Peršič

#### Table S1. Baseline patient characteristics, stratified by mortality.

| Characteristic | survivors, N = 27^1^ | non-survivors, N = 15^1^ | p-value^2^ |
| --- | --- | --- | --- |
| group |  |  | 0.7 |
| Group A | 14 (52%) | 7 (47%) |  |
| Group SOC | 13 (48%) | 8 (53%) |  |
| age (years) | 57 (46, 66) | 66 (53, 70) | 0.12 |
| sex - female | 6 (22%) | 4 (27%) | >0.9 |
| BMI (kg/m^2^) | 31.0 (28.5, 34.1) | 28.4 (26.6, 30.3) | 0.059 |
| diagnose |  |  | 0.7 |
| IHCA | 1 (3.7%) | 0 (0%) |  |
| OHCA | 4 (15%) | 1 (6.7%) |  |
| other | 1 (3.7%) | 2 (13%) |  |
| shock - other | 9 (33%) | 4 (27%) |  |
| shock - sepsis | 12 (44%) | 8 (53%) |  |
| ECMO | 8 (30%) | 2 (13%) | 0.3 |
| RRT | 27 (100%) | 15 (100%) |  |
| MAP (mmHg) | 55 (50, 64) | 49 (48, 59) | 0.12 |
| SAPS II | 83 (72, 92) | 83 (75, 93) | 0.6 |
| noradrenaline (mg/kg/h) |  |  |  |
| T0 | 0.04 (0.01, 0.04) | 0.03 (0.02, 0.05) | 0.8 |
| 4h | 0.04 (0.02, 0.05) | 0.04 (0.03, 0.07) | 0.4 |
| 8h | 0.03 (0.02, 0.05) | 0.05 (0.03, 0.08) | 0.093 |
| 12h | 0.03 (0.02, 0.05) | 0.05 (0.04, 0.09) | 0.10 |
| vasopressin | 5 (19%) | 5 (33%) | 0.5 |
| adrenaline | 0 (0%) | 1 (6.7%) | 0.4 |
| dobutamine | 9 (33%) | 4 (27%) | 0.7 |
| any additional vasopressor | 11 (41%) | 7 (47%) | 0.7 |
| lactate, T0 (mmol/L) | 5.1 (3.1, 8.2) | 7.8 (4.6, 12.5) | 0.2 |
| lactate, 12h (mmol/L) | 3.6 (2.5, 7.6) | 12.5 (7.0, 14.8) | **<0.001** |
| procalcitonin, T0 (µg/L) | 16 (5, 46) | 11 (2, 41) | 0.5 |
| procalcitonin, 12h (µg/L) | 20 (8, 55) | 12 (9, 22) | 0.2 |
| CRP, T0 (mg/L) | 184 (94, 290) | 167 (48, 195) | 0.3 |
| CRP, 12h (mg/L) | 230 (150, 377) | 133 (60, 222) | **0.027** |
| IL-6, T0 (ng/L) | 1,967 (280, 6,120) | 29,896 (7,938, 133,248) | **0.002** |
| IL-6, 12h (ng/L) | 700 (125, 2,312) | 16,135 (1,876, 36,818) | **0.002** |

^1^n (%); Median (IQR)

^2^Chi-squared test; Wilcoxon rank sum test

^3^ eGF – estimated glomerular filtration, CKD-EPI Creatinine 2021

ECMO – extracorporeal membrane oxygenation, RRT – renal replacement therapy (in 12h), MAP – mean arterial pressure, SAPS II – simplified acute physiology score 2

#### Table S2. Noradrenaline requirement (in mg/kg/h), compared to T0, stratified by patient groups.

| group^1^ | time | p-value^2^ |
| --- | --- | --- |
| adsorber | 4 h | 0.052 |
| adsorber | 8 h | 0.237 |
| adsorber | 12 h | 0.305 |
| SOC | 4 h | 0.079 |
| SOC | 8 h | 0.170 |
| SOC | 12 h | 0.408 |

^1^ SOC – standard of care

^2^ Wilcoxon rank sum test
